# Melon/cowpea intercropping pattern influenced the N and C soil cycling and the abundance of soil rare bacterial taxa

**DOI:** 10.3389/fmicb.2022.1004593

**Published:** 2022-11-07

**Authors:** Jessica Cuartero, Jose Antonio Pascual, Juana-María Vivo, Onurcan Özbolat, Virginia Sánchez-Navarro, Julia Weiss, Raúl Zornoza, María Martínez-Mena, Eloisa García, Margarita Ros

**Affiliations:** ^1^Centre of Edaphology and Applied Biology of the Segura (CSIC), University Campus of Espinardo, Murcia, Spain; ^2^Department of Statistics and Operations Research, CMN & IMIB-Arrixaca, University of Murcia, Murcia, Spain; ^3^Institute of Plant Biotechnology, Plaza del Hospital s/n, Technical University of Cartagena, Cartagena, Spain; ^4^Department of Agricultural Science, Polytechnic University of Cartagena, Cartagena, Spain

**Keywords:** intercropping, cowpea, melon, 16S rRNA, nitrogen cycle, PICRUSt2, carbon cycle

## Abstract

The high use of pesticides, herbicides, and unsustainable farming practices resulted in losses of soil quality. Sustainable farming practices such as intercropping could be a good alternative to traditional monocrop, especially using legumes such as cowpea (*Vigna unguiculata L. Walp*). In this study, different melon and cowpea intercropping patterns (melon mixed with cowpea in the same row (MC1); alternating one melon row and one cowpea row (MC2); alternating two melon rows and one cowpea row (MC3)) were assayed to study the intercropping effect on soil bacterial community through 16S rRNA region in a 3-year experiment. The results indicated that intercropping showed high content of total organic carbon, total nitrogen and ammonium, melon yield, and bacterial diversity as well as higher levels of beneficial soil microorganisms such a *Pseudomonas, Aeromicrobium, Niastella*, or *Sphingomonas* which can promote plant growth and plant defense against pathogens. Furthermore, intercropping showed a higher rare taxa diversity in two (MC1 and MC2) out of the three intercropping systems. In addition, N-cycling genes such as *nirB, nosZ*, and *amoA* were more abundant in MC1 and MC2 whereas the *narG* predicted gene was far more abundant in the intercropping systems than in the monocrop at the end of the 3-year experiment. This research fills a gap in knowledge about the importance of soil bacteria in an intercropping melon/cowpea pattern, showing the benefits to yield and soil quality with a decrease in N fertilization.

## Introduction

Soil is a diverse, complex, and yet widely unknown ecosystem in the world, but it is crucial for life because it produces more than 98% of human food (Kopittke et al., 2019). That food production is being compromised, however, mainly due to soil degradation and environmental changes, erosion, loss of soil organic ca rbon, loss of soil fertility, nutrient imbalance, acidification, salinization, and loss of microbial diversity, which are some of soil degradation processes (Dai et al., 2018; Zhou et al., 2018; Gedamu, 2020). There are many factors that affect soil degradation, although it is well known that agricultural management is one of the major contributors (Lal, 2015) through the use of fertilizers, pesticides, herbicides, and other types of practices such as tillage or monoculture (Tetteh, 2015). Therefore, one of the greatest challenges of this century is changing these practices into more sustainable ones such as intercropping.

Crops selected for intercropping normally have different abilities to use the resources available for growth, which leads to yield advantages and increased stability of crop yield compared to a single crop in a low input system (Wang et al., 2015; Dong et al., 2018). However, the use of intercrops has largely focused on the cereal-legume combination (Padhi and Panigrahi, 2006; Dwivedi et al., 2015), and there is still a significant lack of knowledge regarding other types of intercropping, especially with *Cucurbitaceae* plants Melon (*Cucumis melo L*.) is a fruit of great importance around the globe Spain is one of the largest melon exporters in the world, and most of these melons are grown in Murcia (OEC, 2019). Intensive melon cultivation can generate soil and water degradation due to the excessive use of pesticides to reduce the impact of pathogens, and to the necessary application of synthetic fertilizers, due to nutrient depletion (Li et al., 2001). Cowpea (*Vigna unguiculata L. Walp*) is a legume adapted to drought stress (Franke et al., 2018) that is frequently used in intercropping systems with maize (*Zea mays L*.), shorghum (*Sorghum bicolor L. Moench*) and pearl millet (*Pennisetum glaucum LRBr*) (Namatsheve et al., 2021). Legumes have a symbiotic relationship with nitrogen-fixing bacteria through the increased abundance of the gene *nifH*. They can capture nitrogen from the air, so intercropping with legumes allows neighboring crops to absorb more nitrogen from the soil, thus constituting a natural form of biofortification (Zuo and Zhang, 2009; Xue et al., 2016). This helps increase the quality of the fruits and soil (Ritchie and Roser, 2017). In addition, previous studies have reported that the planting patterns could also affect the soil and yield (Xianhai et al., 2012; Raza et al., 2019), so it is necessary to study different intercropping as well as plant distribution.

The influence of intercropping on soil microbial communities has been studied in several agriculture systems (Chen et al., 2018; Lian et al., 2019). In an intercropping system, the roots of different plant species interact with each other and subsequently affect root exudation, which undoubtedly alters the microbial diversity, structure, and functionality (Broeckling et al., 2008; Lian et al., 2019) and affects nutrient transport and mineralization (Rashid et al., 2016). The changed microbial community and activity could affect C and N dynamics, probably due to the ability of microbial communities to regulate C and N use efficiency (Mooshammer et al., 2014). Soil microbial communities are highly diverse and contain both abundant and rare taxa that are crucial for regulating multiple soil processes (Fuhrman, 2009; Delgado-Baquerizo et al., 2018). These rare taxa could be key in certain soil functions, and since their numbers are low, small changes in the soil ecosystem can affect them and even cause them to disappear (Zhou and Wu, 2021). Dominant taxa, on the other hand, would need more soil disturbance to vanish Studying the rare taxa could, thus, indicate changes in soil quality. Relatively little is known about how abundant and rare taxa respond to intercropping drivers, or how microbial community drives biogeochemical cycles.

A three-year intercropping experiment (melon/cowpea) with different patterns was conducted to evaluate the impact on the soil bacterial community (abundant and rare taxa), functionality, and associated ecosystems, by analysis of high-throughput sequencing and soil properties. We hoped to find out (a) whether intercropping patterns affect microbial diversity in the same order for overall microbial community, abundant and rare bacterial taxa; (b) if the intercropping patterns influenced the bacterial community composition differently; and (c) whether the intercropped system enhanced soil macronutrients through changes in predicted microbial genes involved in C- and N-cycling.

## Materials and methods

### Experimental design and sampling

The soil used in this study was classified as Haplic Haplic Calcisol (Loamic, hypercalcic) IUSS (IUSS Working Group WRB, 2015) from La Palma, Cartagena, (37° 41′18″N 0° 56′60″ W), a province of Murcia (SE Spain), in May-August 2018 The detailed experiment is related to Cuartero et al. (2022). Briefly, the treatments used were: (i) melon (*Cucumis melo*) monocrop (M); (ii) mixed intercropping, with melon mixed with cowpea in the same row (MC1); (iii) row intercropping at a ratio of 1:1 (melon:cowpea), alternating one melon row and one cowpea row (MC2); and (iv) row intercropping at a ratio of 2:1 (melon:cowpea), alternating two melon rows and one cowpea row (MC3) (Supplementary Figure 1). All crops were drip irrigated and grown under organic management. The melon plot (M) received the equivalent of 3,000 kg ha-1 of organic fertilizer (N org) (3.2% N and 7% K_2_O). The intercropped plots (MC1, MC2, and MC3) received 30% less Norg than the melon monocrop to assess the efficiency of the intercropping in reducing external fertilization needs.

Five random soil subsamples (0–10 cm depth) were collected in the first and third year of the melon/cowpea intercropping treatments corresponding to 10 August 2018 (referred to as first) and 11 September 2020 (referred to as third). Samples were labeled and immediately brought to the lab where they were separated into two aliquots. The soil was sieved through 2 mm mesh, and the major part was stored at −20°C for biological analysis; a sub-sample was air-dried for chemical analyses.

### Soil DNA extraction, PCR amplification, and sequencing

Soil DNA extraction and Next-Generation-Sequencing of bacterial 16S hypervariable regions were performed according to Cuartero et al. (2022). Briefly, soil DNA was extracted from 1 g of soil (wet weight) using DNeasy Power Soil Kit (Qiagen). Quantity and quality of DNA were tested through Qubit 2.0 fluorometer (Invitrogen, Thermo Fisher Scientific, USA) and NanoDrop 2000 (Thermo Fisher Scientific, Waltham, MA, USA). Ion TorrentTM Personal Genome MachineTM (PGM) was employed to amplify 16S hypervariable region using Ion XpressTM Plus Fragment Library Kit in combination with Ion XpressTM Barcode adapter (Thermo Fisher Scientific), the detailed process is described in Cuartero et al. (2022).

### Sequencing data processing

Bacterial raw sequences, barcodes, and primers were trimmed according to the BaseCaller application. The sequences were denoised with ACACIA (Bragg et al., 2012), and imported to QIIME2 v20202 (Bolyen et al., 2019). Then, sequences were denoised using the DADA2 algorithm with sequences truncated with a *Q* >30 (Callahan et al., 2016) on average. Amplicon Sequence Variants (ASV) obtained from DADA2 were classified using the “classify-consensus-vsearch” command against the SILVA 132 (Quast et al., 2012) database. Functional analysis of the bacterial community was carried out using the PICRUSt2 (Phylogenetic investigation of communities by reconstruction of unobserved states) algorithm (Douglas et al., 2020). Some of the predicted functional genes were then selected to study the effects of intercropping on N- and C-cycling in the soil.

The sequences were uploaded to the European Nucleotide Archive (ENA) with the study accession code PRJEB42624.

### Soil properties

The soil pH, Electrical Conductivity (EC), Total Organic Carbon (TOC), Total Nitrogen (TN), and Ammonia (NH${}_{4}^{+}$) were measured according to Cuartero et al. (2022), briefly, EC was measured in deionized water (1:5 w/v), TOC and TN were determined using CHNS-O analyzer (EA-1108, Carlo Erba). NH${}_{4}^{+}$ was extracted with 2M KCL in a 1:10 soil: extractant ratio and measured by colorimetric assay.

### Statistical analysis

Random forest (RF) analysis was used to test the most important microbial taxa across the intercropping systems at the two sampling times by the randomForest package v 4.7-1 (Liaw and Wiener, 2002). Establishing an RF classifier, which contains a multitude of decision trees based on the threshold abundance of the critical genus (Yu et al., 2021). To enhance the RF classification performance, the optimal three RF hyper-parameters were searched through the “train” function from the caret package v 6.0-91 (Kuhn et al., 2020). The number of variables selected as a splitting parameter at each node (mtry), the number of decision trees (ntree), and the maximal number of nodes (maxnodes) in the forest An 18-fold cross-validation was employed to assess the performance of the classification using the “rfcv” function, as suggested by Zhang et al. (2019). All samples (*n* = 20) were used as the training set and RF classification (proximity = TRUE, importance = TRUE). Finally, the “varImpPlot” and “MDSplot” functions were used to show the importance of taxa and performance in classification, respectively Prior to the test, the differences among cropping systems, normality, and homogeneity of variance assumptions were assayed by Shapiro-Wilk and Levene's tests using the package car (Fox et al., 2007). Mean comparisons were performed with a one-way analysis of variance (ANOVA), followed by the *post-hoc* test of Tukey's honestly significant difference (HSD) when the null hypothesis was rejected Non-parametric Kruskal–Wallis test was applied when data did not fit a normal distribution and, if the assayed data were significant, a multiple comparison Z-values test was performed using the “dunnTest” function with Benjamini-Hochberg corrections in the FSA package (Ogle and Ogle, 2017). To test the treatment effects in paired-measures data, the non-parametric rank-based model “nparLD” was performed through the nparLD package v 21 (Noguchi et al., 2012) using f1ldf1 design.

To study the effects of intercropping systems on bacterial community, the ASV data table was split into rare or more abundant taxa being (> 01%) abundant taxa, (< 01%) rare taxa, and total taxa. A Principal Coordinates Analysis (PCoA) was used to visualize the variation of the community composition based on the Bray-Curtis distance. To test the differences between the cropping systems, a Permutational Multivariate Analysis of Variance (PERMANOVA) was conducted using the “betadisper” and “adonis” functions with 999 permutations from the vegan package v 2.5-7 (Oksanen et al., 2020). Alpha diversity as the Shannon index was calculated using the vegan package A Venn diagram was made to study the total ASVs shared among cropping systems Canonical Correspondence Analysis (CCA) using the “cca”, and “envfit” functions from the vegan package was performed to study the correlation of microbial top ten abundance genera with soil properties. A Non-metric Multidimensional Scaling was performed using the “metaMDS” function from the vegan package with the Bray-Curtis distance to visualize the predicted functional genus. All tests were performed using R language (R Core Team, 2020) and plots were made using mainly the ggplot2 package v 3.3.5 (Gómez-Rubio, 2017).

## Results

### Effect of intercropping on physicochemical soil properties and melon yield

The pH and EC were not affected by the interaction between treatment and sampling time (Table 1). The pH diminished significantly in the third year, and it was lower in the intercropped systems (MC1, MC2, and MC3) than in monoculture (M). EC increased significantly in the third year of the experiment, and the highest EC was recorded in MC1. The interaction between the cropping system and sampling time did not affect TOC and TN (Table 1). TOC and TN increased in intercropping systems compared to monoculture and the values were higher in the third year. NH${}_{4}^{+}$ was significantly higher in the intercropped systems than in the monocrop (M) and its levels dropped significantly in the third year (Table 1). The melon yield average was greater in the intercropping systems (MC1, MC2, and MC3) than in the monocrop (M) and was significantly lower value in the third year than in the first year.

**Table 1:** Different soil properties and harvest in different sampling times and cropping systems.

	**First**				**Third**					
	**M**	**MC1**	**MC2**	**MC3**	**M**	**MC1**	**MC2**	**MC3**		***P*-value**
pH	8.48 ± 0.02	8.40 ± 0.02	8.43 ± 0.03	8.42 ± 0.02	7.98 ± 0.07	7.73 ± 0.12	7.83 ± 0.15	7.96 ± 0.06	G T GxT	*** *** ns
EC (dS m^−1^)	0.290 ± 0.001	0.336 ± 0.023	0.297 ± 0.013	0.303 ± 0.028	0.496 ± 0.015	0.555 ± 0.069	0.537 ± 0.040	0.505 ± 0.026	G T GxT	*** *** ns
TOC (g kg^−1^)	9.5 ± 0.1	11.2 ± 0.4	11.1 ± 0.2	11.9 ± 0.2	9.86 ± 0.9	12.3 ± 0.4	11.0 ± 0.6	12.1 ± 1.2	G T GxT	*** * ns
TN (g kg^−1^)	1.10 ± 0.00	1.30 ± 0.00	1.30 ± 0.00	1.30 ± 0.00	1.08 ± 0.04	1.36 ± 0.06	1.34 ± 0.11	1.34 ± 0.06	G T GxT	*** * ns
NH${}_{4}^{+}$ (mg kg^−1^)	0.83 ± 0.13 b	1.83 ± 0.07 a	3.47 ± 0.49 a	4.63 ± 0.56 a	0.76 ± 0.15 b	0.94 ± 0.30 a	0.91 ± 0.19 a	1.06 ± 0.39 a	G T GxT	*** *** ***
Yield (kg ha^−1^)	15,092 ± 230	26,271 ± 3,339	20,287 ± 3,038	24,759 ± 2,049	8,561 ± 428	9,637 ± 2,599	10,600 ± 2,330	10,843 ± 2,451	G T GxT	ns *** ns

(mean±sd; *n* = 5) G, Group (which corresponds to cropping system); *T*, Time; *GxT*, interaction of Group x Time; *M*, Melon monocrop; MC1, Mixed intercropping; MC2, Intercropping row 1:1 melon:cowpea; MC2, Intercropping row 2:1 melon:cowpea; *EC*, Electrical conductivity; *TOC*, Total organic carbon; *TN*, Total nitrogen; NH${}_{4}^{+}$ total ammonium; Yield; Melon Yield Letters represent the significant differences between the cropping system and sampling time. *P* value: *** < 0.001; ** < 0.01; * < 0.05.

A canonical correspondence analysis at both sampling times (Supplementary Figure 2) revealed the influence of soil properties on the microbial community at the genus level. Constrained CCA explained 85 and 60% of the inertia for the first and third years of the experiment, respectively. The top ten genera were highly affected by NH${}_{4}^{+}$, EC, pH, TN, and TOC (*p*-value: 0.002, 0.044, 0.003, 0.001, and 0.001, respectively), in the first year of the experiment, but by the third year of the experiment, they were only significantly affected by pH, TOC, EC and TN (*p*-value: 0.029, 0.011, 0.061, and 0.002, respectively).

### Effect of intercropping on whole, abundant, and rare bacterial diversity

At both sampling times, the different intercropping had a total of 2,140,702 reads ranging from ≈ 28,000 to 66,000 reads per sample which had a total of 9,558 different ASV ranging from 217 to 1,025 ASV per sample. Data were rarefied to 27,978 reads and the rarefaction curve constructed by randomly selected sequences from samples indicating that the number of sequences was representative of the bacterial community (Supplementary Figure 3).

**Figure 1:**
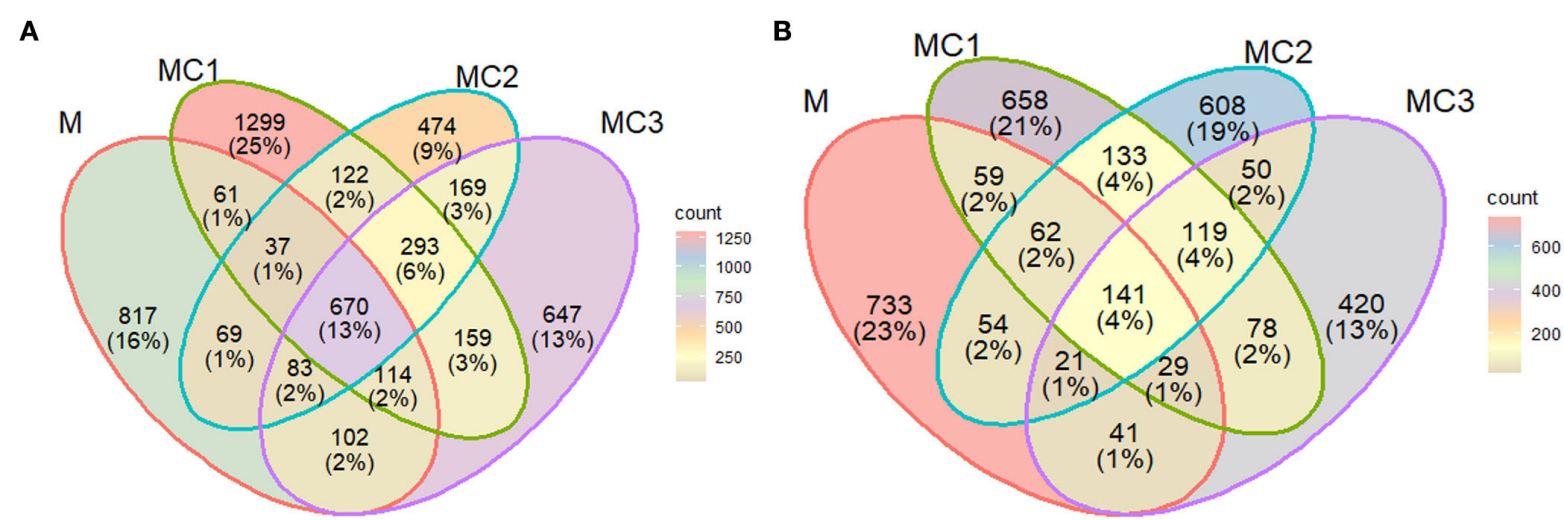
Venn diagram based on ASVs at the first **(A)** and third **(B)** year of the experiment. M, melon monocrop; MC1, mixed intercropping; MC2, intercropping row 1:1 melon:cowpea; MC3, intercropping row 2:1 melon:cowpea. The numbers (percentages) of the abundant bacteria in different systems are indicated in the overlapping and non-overlapping areas.

The ASV obtained were classified into 31 phyla, 394 families, and 638 genera. A Venn diagram showed that all the cropping systems shared 670 (13%) and the intercropped systems 119 (4%). ASV respectively in the first year of the cropping system (Figures 1A,B). However, this number decreased to 141 ASV (4%) for all crop systems and 119 (4%) between intercropped systems in the third year of the cropping system (Figures 1A,B). In general, the effect of intercropping systems showed large differences with monocrop, where MC1 and MC2 shared with monocrop (M) only 1 and 2% of ASVs respectively in both samplings, and MC3 shared with monocrop around 2 and 1% of ASVs in both samplings (Figures 1A,B).

Large differences were found in the ASVs counts from different cropping systems. The ASV data table was split into (> 0.1%) abundant taxa, (< 0.1%) rare taxa, and total taxa. In the first year, intercropping systems (MC1, MC2, and MC3) showed a higher composition of rare taxa (ranging from 5,107 to 6,020%) than the most abundant taxa (ranging from 3,980 to 4,983%). Compared to the monocrop, the intercropping MC3 showed the highest number of total ASVs, and rare taxa (897/540) followed by MC1 (851/490) and MC2 (748/382) respectively, compared to monocrop (M) (Supplementary Table S1). However, in the third year of the experiment intercropping system showed a higher proportion of most abundant taxa (76.61–62.17%) compared to rare taxa (23.39–35.44%), MC2 showed the highest values of total ASVs, and rare taxa (415/157) followed by MC1 (364/129) (Supplementary Table S1).

**Figure 2:**
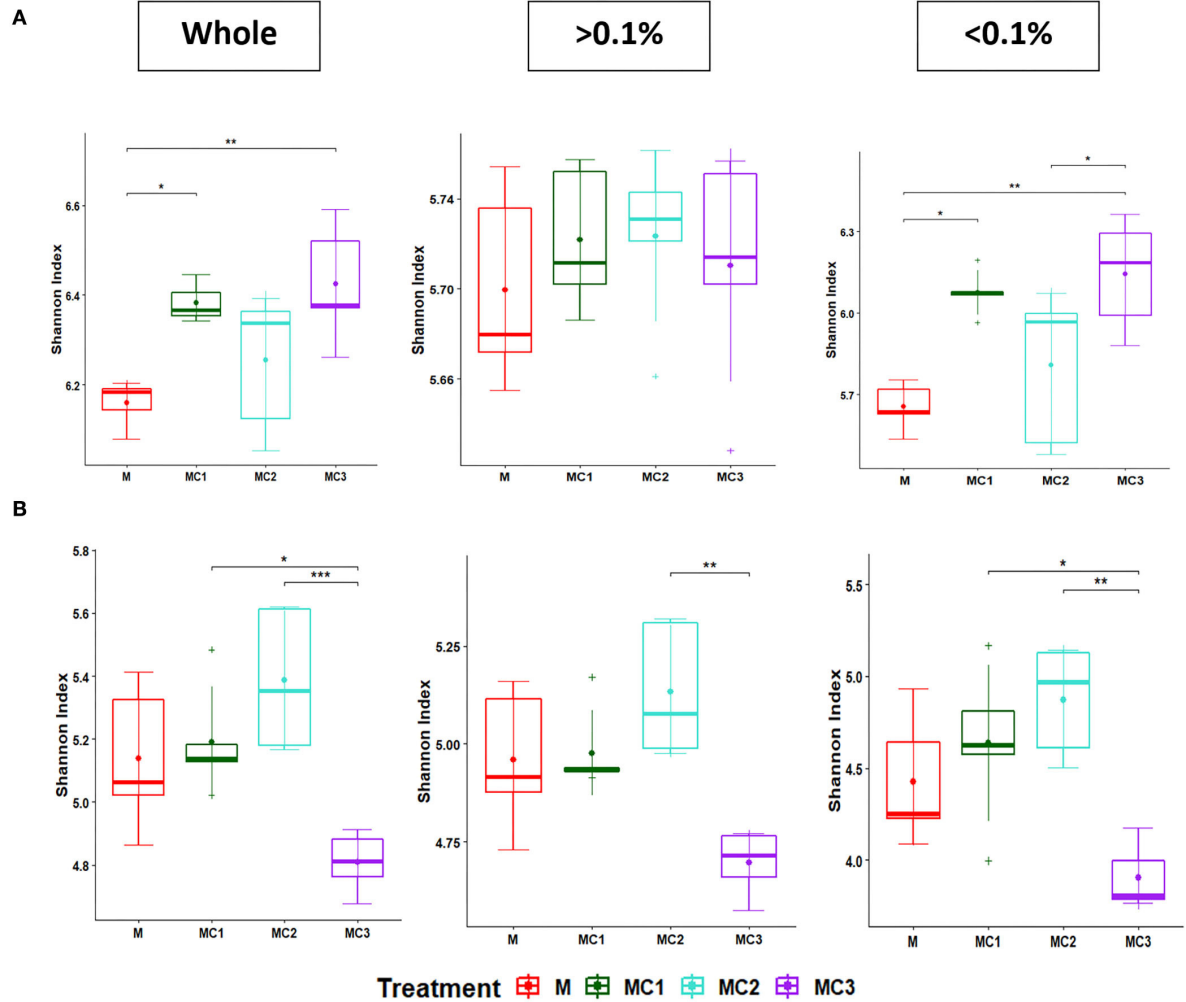
Boxplot of Shannon diversity index of the whole, more abundant, and rare bacterial taxa at the first **(A)** and third **(B)** years under the cropping systems. The “•” and line inside the box plot respectively represent the mean and median, (*n* = 5). “+” outside the box plot are outliers. Significant differences between cropping systems (Kruskal–Wallis test followed by *post-hoc*) are indicated by “*”, < 0.05: “**”, < 0.01; “***”, < 0.001. The “> 0.1%” represents the abundant taxa; “ < 0.1%” represents the rare taxa; whole, total. M, melon monocrop; MC1, mixed intercropping; MC2, intercropping row 1:1 melon:cowpea; MC3, intercropping row 2:1 melon:cowpea.

The Shannon index for the total, most abundant, and rare taxa diminished in the third year (Figure 2; Supplementary Table 2). In the first year of the cropping system, MC1 and MC3 showed the highest total and rare taxa diversity compared to monocrop (M) (Figure 2). In the third year, however, MC1 and MC2 showed higher values than the monocrop (M) while MC3 showed the lowest Shannon index (Figure 2, Supplementary Table 2).

**Figure 3:**
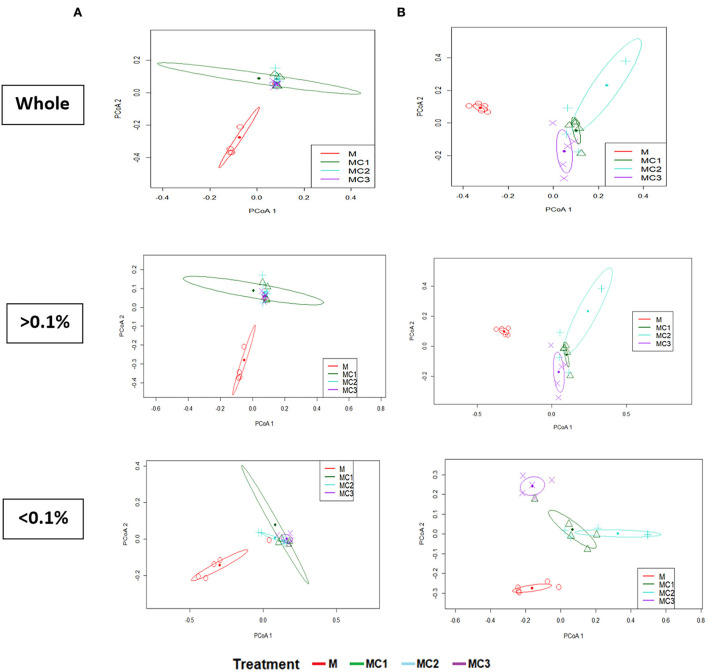
Principal Coordinate Analysis of soil bacteria community (β-Diversity) using Bray-Curtis distance for whole, most abundant, and rare taxa at the first **(A)** and the third **(B)** year under the cropping systems. > 0.1% represents the abundant taxa; < 0.1 represent the rare taxa; whole, total, M, melon monocrop; MC1, mixed intercropping; MC2, intercropping row 1:1 melon:cowpea; MC3, intercropping row 2:1 melon:cowpea.

The β-microbial diversity represented by PCA analysis of the whole, most abundant, and rare taxa at both sampling times showed significant changes (PERMANOVA; *P* < 0.05) in the intercropped treatments compared to the monocrop (M) (Figure 3). In the first year of the experiment, no significant differences were observed between intercropped systems in any of diversity indices, while in the third year, only the rare taxa showed significant differences between the intercropped systems, with MC3 showing different values from MC1 and MC2 (Figure 3).

### Effect of intercropping on responsive bacterial genera

**Figure 4:**
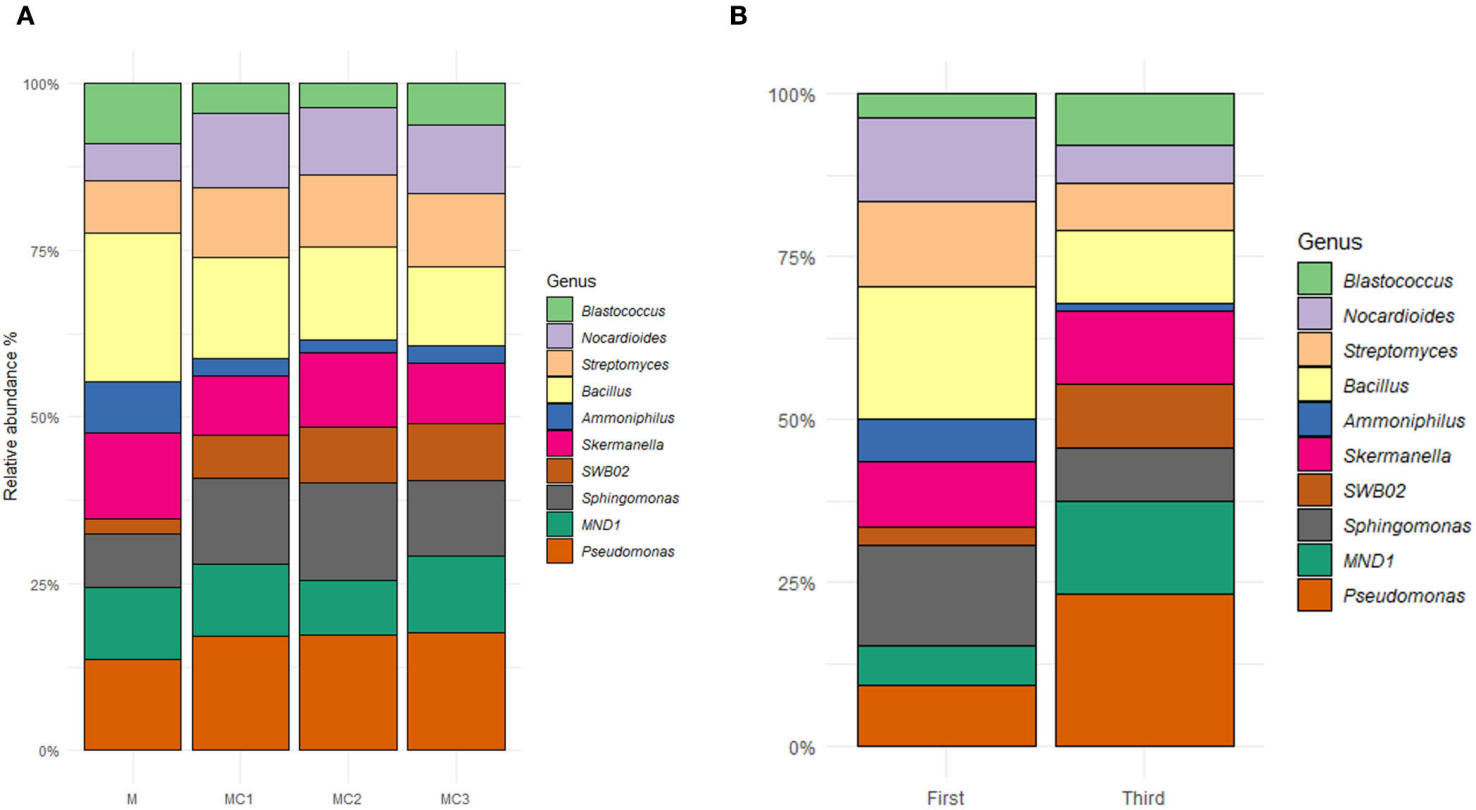
Top ten more abundant genera in **(A)** different cropping systems and **(B)** different sampling times. M, melon monocrop; MC1, mixed intercropping; MC2, intercropping row 1:1 melon:cowpea; MC3, intercropping row 2:1 melon:cowpea; Initial, the first year; final, the last year.

The top 10 most abundant genera cover ≈70% of the evaluated taxa, being identified as *Pseudomonas* (11.60%), *Bacillus* (9.45%), *Sphingomonas* (8.26%), *Skermanella* (7.46%), *MND1* (7.28%), *Streptomyces* (6.97%) *Nocardioides* (6.52%), *SWB02* (4.51%) *Blastococcus* (4.12%) and *Ammoniphilus* (2.66%); all were affected by the type of cropping systems and sampling times (Figure 4, Supplementary Table 3). The relative abundance of *Pseudomonas, Sphingomonas, Nocardioides, SWB02*, and *Streptomyces* was significantly higher in the three intercropped systems, whereas, the relative abundance of *Bacillus, Skermanella, Ammoniphilus*, and *Blastococcus*, was significantly lower compared to monocrop (M) (Figure 4A). Differences were also observed by sampling time, in which *Pseudomonas, Skermanella, MND1, SWB02*, and *Blastoccus* showed higher relative abundance in the third year of the experiment; whereas the relative abundance of *Bacillus, Sphingomonas, Nocardioides, Amoniphilus*, and *Streptomyces* decreased (Figure 4B).

To identify the representative microbes in the cropping systems, random forest analyses with all genera were conducted at both sampling times to find the most important genera (Supplementary Figures 4A,B). The MDS showed how all cropping systems at both sampling times can be clearly differentiated by the selected genera (Supplementary Figures 4C,D). Of the 15 most important genera in the first year of the experiment, *Microvirga, Nonomuraea, Micromonospora, Kribella, Roseomonas*, and *Rubrobacter* were highly enriched in monocrop (M); *Mesorhizobium, Gemmata, SWB02, Iamia*, and *Pontibacter* were highly enriched in MC1 and *Nocardioides, Agromyces, Aeromicrobium*, and *Hypomicrobium* were highly enriched in MC3 (Supplementary Figure 4A). In the third year of the experiment, however, different genera were observed for cropping system, in which *Ammoniphilus, Lysobacter*, and *Bradyrhizobium* were highly enriched in the monocrop (M), *Thauera, Nannocystis, Aeromicrobium, Nonomuraea*, and *Sphingomonas* were highly enriched in MC1; *SM1A02, Niastella, Ensifer*, and *Bryobacter* in MC2, and *Vogesella, Streptomyces*, and *Policyclovorans* were highly enriched in MC3 (Supplementary Figure 5B).

### Effect of intercropping systems on predictive functional profiling of soil microbial communities

To understand the phylogenetic or taxonomic composition of bacterial communities, it is necessary to study the predictive community functions of 16S rRNA sequencing data. Most pathways did not show the interaction between cropping systems and sampling time except for Citrate Cycle (TCA) (Supplementary Table S4). Bacterial secretion, nitrogen metabolism, energy metabolism, and transporters increased significantly in the third year, whereas the TCA cycle and protein export decreased. In general, all the intercropped systems showed higher values of the different studied pathways compared to monoculture (M) showing the highest values in MC1 and MC2 (Supplementary Table S4).

### Functional predicted N-cycling genes

In general, the predicted N-Cycling genes were highly affected by the cropping system, sampling time, and their interaction (Supplementary Table S5). In the third year, potential nitrogen fixation—identified by *nifT* and *nifX* genes—was highest in MC1 and was significantly lower in MC2 and MC3 than in the monocrop (M) (Supplementary Table S5). Dissimilatory nitrogen reduction genes (*narG* and *nirB*) involved in the denitrification process were significantly more abundant in the third year and showed higher values in the intercropping systems, among which MC1 and MC2 showed the highest values (Supplementary Table S5). The abundance of *amoA* and *amoC* genes involved in the nitrification process was higher in the first year than in the third. At the end of the experiment, MC2 showed the highest values. The predicted gene *nosZ* showed higher values in the third year than in the first year For this gene, the intercropping systems had significantly higher values than the monocrop (M) and MC1 and MC2 showed the highest values (Supplementary Table S5).

**Figure 5:**
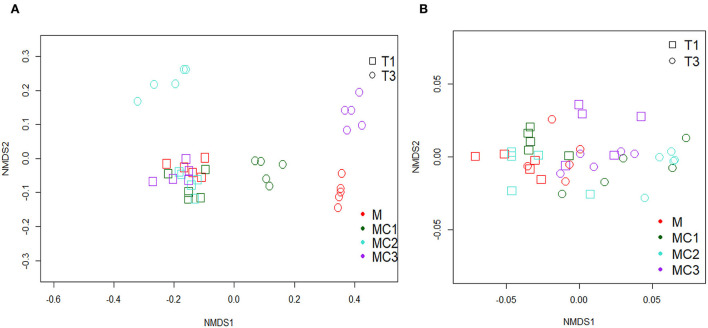
A Non-metric Multi-dimensional Scaling (NMDS) of predicted nitrogen cycle genes **(A)** and predicted carbon cycle genes **(B)** M, melon monocrop; MC1, mixed intercropping; MC2, intercropping row 1:1 melon:cowpea; MC3, intercropping row 2:1 melon:cowpea; T1, First year; T3, Third year.

The NMDS plot of predicted N-Cycling genes showed no observable differences among cropping systems in the first year (Figure 5A). This trend changed in the third year of the experiment, however, when the cropping systems were clearly differentiated (NMDS Stress = 0.039).

### Functional predicted C-cycling genes

To evaluate the carbon availability to soil bacteria under different cropping systems, the predicted genes *glcD* (Glycolate oxidase subunit), *xylA, alpha-amylase, glucokinase*, and *pyruvate kinase* were studied. The results showed that all genes were highly affected by the cropping system, sampling time, and their interaction (Supplementary Table S6). All predictive genes increased in the third year of the experiment compared to the first year (Supplementary Table S6). In the first year, the highest values were found in MC3 followed by MC1 and MC2 compared to Monocrop, while, in the third year the highest values were found in MC1 and MC2 compared to monocrop (M) (Supplementary Table 6). NMDS from C-Cycling predicted genes (Figure 5B) showed that predicted carbon genes clustered together (NMDS Stress = 0.08) in all treatments at different times.

## Discussion

Intercropping is an environmentally friendly method that plays an important role in improving soil quality and in controlling pests and disease occurrence (Dai et al., 2019). The degree of improvement depends on the crop species, the intercropping combinations, and their spatial distribution (Raseduzzaman and Jensen, 2017; Ren et al., 2017; Zhang et al., 2018b; Salgado et al., 2021). Studies are scarce, however, concerning melon/cowpea intercropping and patterns Our results revealed that the bacterial communities were altered by the intercropping system and pattern, a fact that is attributed to the root interactions between the different plant species, which subsequently affects root exudation (Broeckling et al., 2008; Lian et al., 2019). This undoubtedly alters the microbial diversity, structure, and functionality (Li and Wu, 2018; Zeng et al., 2019). Bacteria have been linked to C acquisition strategies and their interactions can contribute to soil stabilization through the biopolymers they exuded (Chenu, 1989; Deng et al., 2015) which act as binding agents in which bacteria are causal factors in enhancing soil aggregation (Lehmann et al., 2017). It highlighted the changes in those members contributing to the “rare biosphere” (Pascoal et al., 2021) increasing the sensitivity of detecting effects on microbiomes as having been observed by other studies (Jiao and Lu, 2020a; Ji et al., 2020), where they suggested that abundant taxa play a dominant role in the stability and maintenance of agro-soils whereas rare taxa have a high influence under environmental disturbances (Jiao and Lu, 2020b). In our study, the increase in bacterial diversity (Supplementary Table S1, Figure 2) was due principally to the increase of rare bacterial diversity (oligotrophic and synergistic bacteria) instead of the most abundant (copiotrophics and competitive) bacteria, as was observed by Liu et al. (2022). It is possible that rare bacteria had to perform synergistic activities for extracting energy and carbon from more complex organic substrates during the 3-year experiment. This would enable rare bacteria community members to carry out more elaborated interactions than the abundant bacteria, which get their nutrients and energy from roots, whose exudates would promote competition between fast-growing copiotrophs (Fierer et al., 2012; Liu et al., 2022). Rare taxa could have a more important role in functionality, which can help to crop growth (Chen et al., 2020; Liang et al., 2020; Xiong et al., 2021). The Bacterial diversity of this study could be affected after 3 years of intercropping by the decrease in pH since the acidification can change the stability of bacterial cell membranes and thus inhibits bacterial growth (Feng et al., 2014; Zarafshar et al., 2020). Beta diversity—which represents the extent of change in community composition, or the degree of community differentiation—showed differences between monocrop (M) and intercropped systems (Figure 3) in whole bacteria and the most abundant taxa However, in the third year, if we focus only on the rare taxa, beta diversity showed differences according to the pattern—MC1 and MC2 grouped separately from MC3 and M. This could indicate that higher plant biomass in MC1 and MC2 induce higher soil organic matter fraction levels, intercropped systems can induce higher soil organic matter fraction contents which could act as major sources of energy for microorganisms (Tian et al., 2013).

The Intercropping systems showed an increase in beneficial microorganisms (*Pseudomonas, Sphigomonas, Streptomyces, Nocardioides*, and *SWB02*) (Supplementary Table S3, Figure 4), as other authors have observed in different intercropping systems (Yu et al., 2019; Zhao et al., 2022) *Sphingomonas* promote nitrogen fixation and dehydrogenation (Leys et al., 2004) and has also been found to increase plant growth hormone production (Asaf et al., 2020). Some species of *Pseudomonas* can enhance nutrient uptake and thus plant growth (Franke-Whittle et al., 2015; Lami et al., 2020) *SWB02*, on the other hand, has been linked to the capacity to oxidize nitrite to nitrate (Fumasoli et al., 2015; Gao et al., 2021), while *Niastella* (Supplementary Figure 4B) can mitigate N_2_0 emissions (Nishizawa et al., 2014) *Aeromicrobium* (Supplementary Figures 4A,B) has been linked to plant biomass (Nuzzo et al., 2020) and has a greater capability to produce secondary compounds with antimicrobial capacities (Miller et al., 1991). These bacteria were highly correlated with TN, TOC, and pH at both sampling times (Supplementary Figure 2). Cong et al. (2015) observed that the increases in aboveground productivity by enhancing soil C and N should occur due to the microbial species complementarity, as our results showed (Supplementary Figure 2).

Nitrogen is an essential element for plant growth and development, and it is the element most closely related to yield (Zhao et al., 2022). Our results showed significantly higher TN and NH${}_{4}^{+}$ (Table 1) in intercropped than in the monocrop (M), even considering that 30% less fertilization was used in the intercropped systems than in the monocrop. An increase in melon yield was observed in intercropping systems at both sampling times. In previous studies, Cuartero et al. (2022) observed higher yield in intercropped systems probably due to low N fertilizer addition (Yu et al., 2018). This fact has previously been observed in other cowpea intercrop relationships, such as cowpea-maize (Latati et al., 2014), cowpea-sorghum (Oseni, 2010), and cowpea-cassava (Sikirou and Wydra, 2008). Also, this finding may be due to the complex biological diversity under intercropping systems that resulted in the transfer of N to soil *via* ions and root exudates and further facilitated the accumulation and decomposition of soil N fractions. In legume-mixed intercropping, legumes increase N_2_ fixation, thus providing higher N levels for the adjacent crop, thus yielding a growth advantage for the intercropped plant (Subedi and Ma, 2005; Yu et al., 2018). In addition, the higher plant litter and root exudates by intercropping compared to monoculture may have been responsible for the increase in TOC content (Table 1). Castellano et al. (2015) suggested that plant litter is the primary source of all SOM. No differences were observed between intercropped systems probably due to the use of only one intercrop species because according to Zhang et al. (2021) different intercropped resulted in differences in the N cycle predicted genes, and these differences might be caused by variations in the quantity and quality of plant litter and root exudates among intercrops.

Biological factors, such as microorganisms, could indicate the environmental balance through biotic indexes derived from the observation of taxa. Therefore, in this study, random forest analysis (Supplementary Figure 4) identified specific genera as possible indicators of different intercropping systems, where only 13% of the genera belonged to the most abundant genera in intercropping systems, and the sampling time showed a stronger influence of these biomarkers than the different intercropping patterns.

When a bacterial community structure is altered due to a disturbance (Figure 3), as in this study (intercropping), functional redundancy (an overlap in the ecological functions of various species) is very important for maintaining the functionality of the community (Wohl et al., 2004; Comte and del Giorgio, 2010; Baho et al., 2012) and this fact does not produce a relationship between disturbance and microbial structure. However, our results (Supplementary Table S4) indicated an increase of prediction functional genes principally in MC1 and MC2, such as bacterial secretion system, protein export, transporter, and energy metabolism that could indicate a higher activity of soil bacteria and a higher secretion of protein and agents promoting soil aggregation (Oliveira et al., 2019). Also, an increase in carbon fixation and the TCA cycle and N metabolism could indicate an acceleration of nutrient conversion (Shi et al., 2017). The TCA cycle, also known as the Krebs cycle and citric cycle, is the main source of energy for cells and essential for aerobic respiration to deal with oxidative stress and produce energy for secreting defense compounds (Zhang et al., 2018a).

Six prediction genes (Supplementary Table S5) involved in different steps of the Nitrogen cycle showed, by NMDS (Figure 5A), differences between cropping systems in the third year of the experiment which could indicate that although bacterial abundance and diversity diminished this cycle is not N-limited and each intercropped system have system-specific bottlenecks in the N cycle N_2_ fixation gene expression is strongly dependent on the level of mineral N in soil (Li et al., 2016). Intercropping system with cowpea in the third year showed, in general, higher nitrogen fixation genes principally in MC1 with less inorganic fertilization than melon monocrop (M). Root-derived compounds may induce nodulation *via* hormone signaling and stimulate N_2_ fixation by increasing the activities of proteins involved in N_2_ fixation at the gene expression and physiological levels (Li et al., 2016). However, more than half of the increased N_2_ fixation under intercropping could be also attributed to soil micro-organisms including members of the phyla Actinobacteria (*Arthrobacter* and *Agromyces*), Bacteroidetes, Firmicutes (*Bacillus* and *Psychrobacillus*) and Proteobacteria (Rilling et al., 2018) some of them observed in our intercropping systems. Ammonia, which is considered a regulatory signal of symbiosis in the nodules (Patriarca et al., 2002), could be one of the different ways to carry the nitrogen captured in nodules to the rhizosphere of melon since ammonia can diffuse in soil due to its positive charge then it could be oxidized again by nitrifying bacteria like *SM1A02* or *Sphingomonas* (Xie and Yokota, 2006) or uptake by plants.

Genes involved in the denitrification process were higher than nitrification in all cropping systems. Denitrification is the process of dissimilatory reduction of nitrate and nitrite, producing gaseous end products of nitric oxide (NO), N_2_O, and dinitrogen (N_2_). The process of denitrification relies on a series of enzymes that were produced when *narG* and *nirB* genes are expressed. They were highly increased in intercropping systems principally MC1 and MC2 indicating higher emission of N_2_O from soil (Shaw et al., 2006), probably due to excess inorganic fertilization being incorporated However, the increase of gen *nosZ* could indicate the greater conversion of N_2_O–N_2_ and decreased greenhouse gas production (Krause et al., 2017). Nitrification is a biochemical process important for soil fertility in which nitrifying bacteria transform the ammonium into nitrates (NO_3_) to be used by plants (Kant, 2018), where gen as *amoA* and *amoC* are involved and MC1 and MC2 showed the highest values.

In addition, a higher content of alpha-amylase, xylose, and glycolate oxidase (Supplementary Table S5) abundance potential genes (belonging to carbon and glycolate cycle) related mainly with *Bacillus, Caulobacter, Streptomyces*, and other decomposers genera (Wijbenga et al., 1991; Stephens et al., 2007; Gubbens et al., 2017; Luang-In et al., 2019; Ibrahim et al., 2021) could help to increase the availability of nutrients for plants Fernández-Bayo et al. (2019) showed that organic exudates from the rhizosphere can increase these C degrading pathways. According to these results, intercropping systems MC1 and MC2 have a greater diversity of energy and carbon sources so cowpea could lead to a more complex and capable microbial community than traditional monoculture, as it was pointed out by Li et al. (2022). Carbon fixation was also enhanced (Supplementary Table S4) in soils with higher cowpea plants density which is commonly incorporated into soils through microorganisms (Berg et al., 2010) and is one of the most important steps in the carbon cycle, a crucial step for CO_2_ assimilation and sequestration and it has been previously related with soils with high bacteria diversity (Lynn et al., 2017).

## Conclusion

The study of beta diversity using the rare taxa instead of the whole taxa was able to show differences between intercropped patterns (MC1 and MC2 compared to MC3). Intercropping systems showed higher value content of total organic carbon (TOC), total nitrogen (TN), melon yield, and bacterial diversity as well as a higher content of beneficial soil microorganisms such as *Pseudomonas, Aeromicrobium, Niastella* or *Sphingomonas* which can promote plant growth and its protection against different pathogens. Predictive N and C cycling genes showed higher abundance in the intercropped system than in monocrop, and also showed differences between intercropped systems, where MC1 and MC2 showed higher abundance than MC3.

## Data availability statement

The datasets presented in this study can be found in online repositories. The names of the repository/repositories and accession number(s) can be found in the article/Supplementary material.

## Author contributions

Conceptualization: JC, VS-N, RZ, JW, JP, J-MV, MM-M, EG, and MR. Methodology: JC, OÖ, VS-N, JW, RZ, JP, J-MV, EG, MM-M, and MR. Validation: RZ, JP, and MR. Formal analysis and data curation: JC and J-MV. Investigation and resources: JC, OÖ, VS-N, JW, RZ, JP, J-MV, and MR. Writing—original draft preparation: JC. Writing—review and editing and supervision: JP, J-MV, and MR. Visualization: JC and J-MV. Project administration and funding acquisition: JP and MR. All authors have read and agreed to the published version of the manuscript.

## Funding

This study was supported by the AsociaHortus project [AGL2017-83975-R] funded by the Spanish Ministry of Science, Innovation and Universities, and the European Commission Horizon 2020 project Diverfarming [Grant agreement 728003].

## Conflict of interest

The authors declare that the research was conducted in the absence of any commercial or financial relationships that could be construed as a potential conflict of interest.

## Publisher's note

All claims expressed in this article are solely those of the authors and do not necessarily represent those of their affiliated organizations, or those of the publisher, the editors and the reviewers. Any product that may be evaluated in this article, or claim that may be made by its manufacturer, is not guaranteed or endorsed by the publisher.
