# Supplementary material for: Melon/cowpea intercropping pattern influenced the N and C soil cycling and the abundance of soil rare bacterial taxa
Source: Front Microbiol. 2022 Nov 7;13:1004593. doi: 10.3389/fmicb.2022.1004593 (PMC9676475; doi:10.3389/fmicb.2022.1004593)
Supplement: Supplementary file 1 [file Data_Sheet_1.docx]

**Supplementary Fig. 1. Plant frame of melon and cowpea distribution in Cuartero et al (2022)**

**
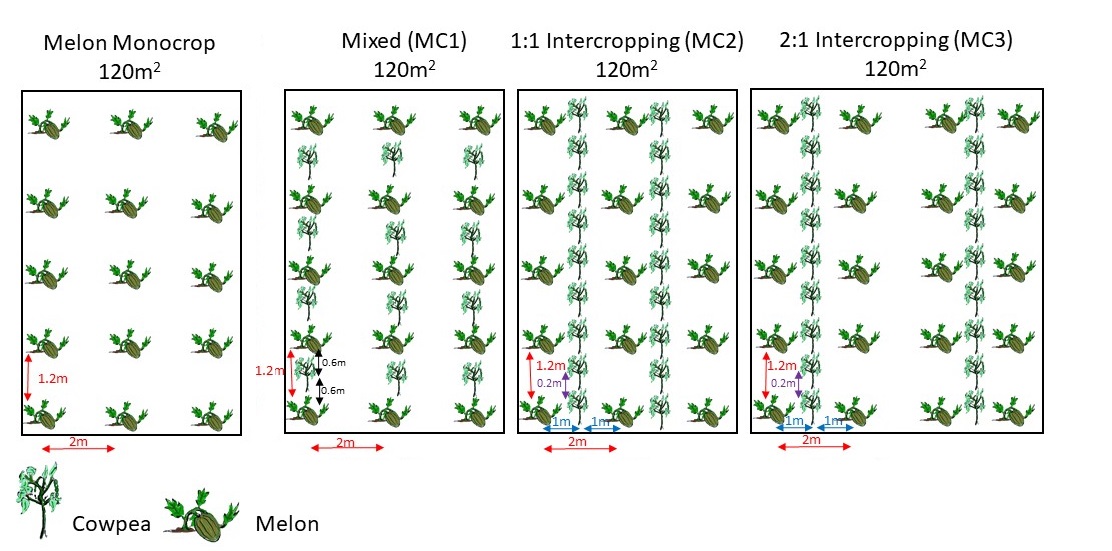
**


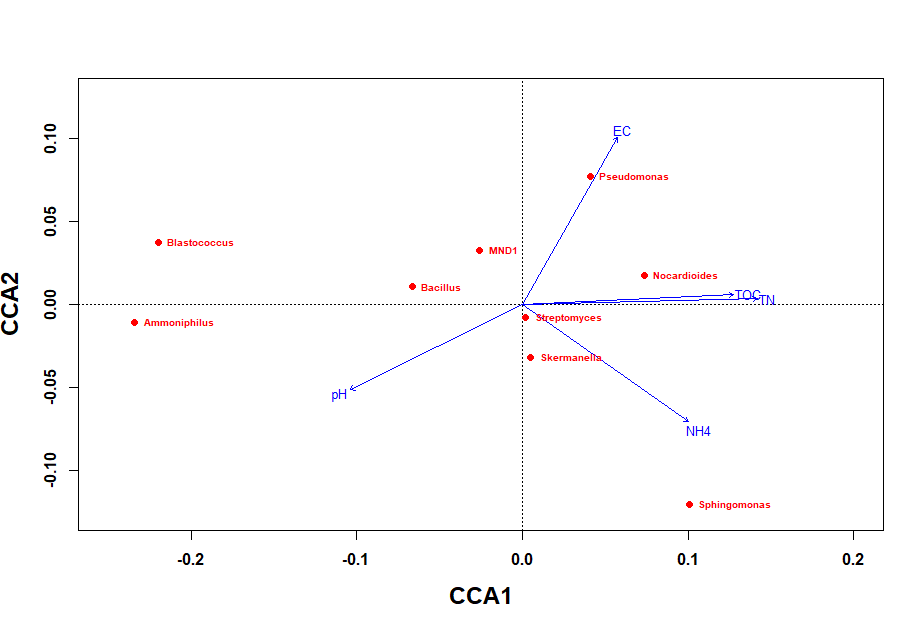


A

B


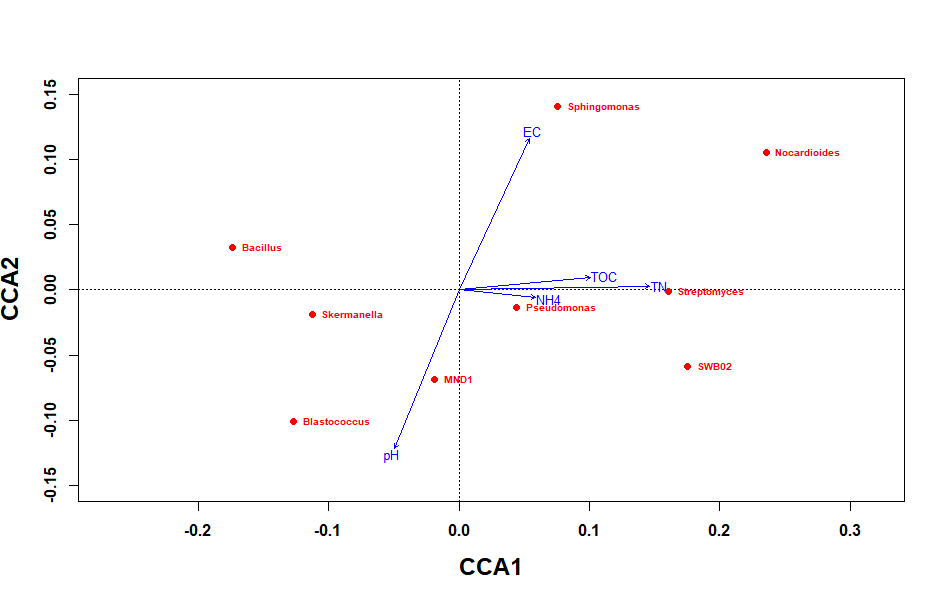


**Supplementary Fig. 2. Canonical Correspondence Analysis (CCA) of soil properties and top ten genera at (A) first at (B) third year of the cropping systems.** Blue arrows represent soil properties, and the red names correspond to top ten taxa at genus level. EC, Electrical conductivity; TOC, Total organic carbon; TN, Total nitrogen; NH_4_^+^, Total ammonium.

**
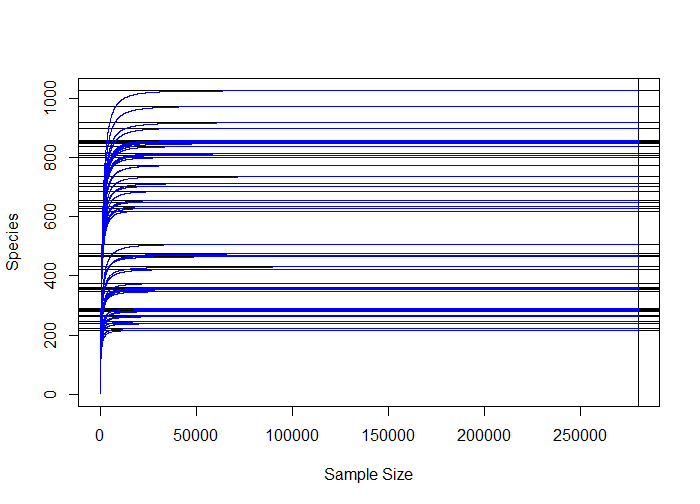
**

A

B

**Supplementary Fig. 3. Rarefaction curve of bacterial community in the first (A) and third (B) year of the cropping systems.**

**Supplementary Table S1. ASVs counts from total, most abundant (> 0.1%) and rare (< 0.1%) taxa in different sampling times and cropping systems.**

| **Year** |  | **M** | **MC1** | **MC2** | **MC3** | **P-Value** |
| --- | --- | --- | --- | --- | --- | --- |
| **First** | Total | 677 c | 851 ab | 748 bc | 897 a | ** |
|  | > 0.1% | 358 | 361 | 366 | 357 | ns |
|  | < 0.1% | 319 c | 490 ab | 382 bc | 540 a | ** |
| **Third** | Total | 324 ab | 364 a | 415 a | 248 b | ** |
|  | > 0.1% | 223 ab | 235 a | 258 a | 190 b | *** |
|  | < 0.1% | 102 ab | 129 ab | 157 a | 58 b | ** |

M; Melon monocrop, MC1; Mixed intercropping, MC2; Intercropping row 1:1 melon:cowpea, MC3; Intercropping row 2:1 melon:cowpea, letters represents the values of pairwise test.

**Supplementary table S2. Shannon diversity index in different sampling times and cropping systems.**

| **Year** |  | **M** | **MC1** | **MC2** | **MC3** | **P-Value** |
| --- | --- | --- | --- | --- | --- | --- |
| **First** | Total | 6.16±0.05 b | 6.38±0.04 a | 6.26±0.15 ab | 6.42±0.13 a | ** |
|  | > 0.1% | 5.70±0.04 | 5.72±0.03 | 5.72±0.03 | 5.71±0.05 | ns |
|  | < 0.1% | 5.65±0.09 c | 6.08±0.08 ab | 5.81±0.28 bc | 6.14±0.20 a | ** |
| **Third** | Total | 5.14±0.22 ab | 5.19±0.17 a | 5.39±0.22 a | 4.81±0.09 b | ** |
|  | > 0.1% | 4.96±0.18 ab | 4.98±0.11 ab | 5.13±0.17 a | 4.70±0.08 b | ** |
|  | < 0.1% | 4.43±0.34 ab | 4.64±0.43 a | 4.87±0.30 a | 3.91±0.18 b | ** |

(mean±sd; n=5), M; Melon monocrop, MC1; Mixed intercropping, MC2; Intercropping row 1:1 melon:cowpea, MC3; Intercropping row 2:1 melon:cowpea, letters represents the values of pairwise test.

**Supplementary Table S3.** **Relative abundance of top 10 most abundant genera**

|  | **M** | | **MC1** | | **MC2** | | | **MC3** | | |  |  |
| --- | --- | --- | --- | --- | --- | --- | --- | --- | --- | --- | --- | --- |
|  | **First** | **Third** | **First** | **Third** | **First** | | **Third** | **First** | **Third** | | **P-Value** | |
| ***Pseudomonas*** | 5.54±0.35 | 14.15±4.35 | 8.57±1.61 | 13.94±0.63 | 5.06±0.93 |  | 20.06±2.78 | 6.42±1.09 | | 18.99±2.56 | **G**  **T**  **Gx** | **  ***  *** |
| ***Bacillus*** | 17.71±2.10 | 14.73±2.36 | 12.98±1.04 | 6.78±0.84 | 13.37±0.74 |  | 6.78±0.98 | 12.09±0.87 | | 4.73±0.89 | **G**  **T**  **Gx** | ***  ***  ** |
| ***Skermanella*** | 6.80±0.54 | 11.87±0.90 | 5.75±0.73 | 5.83±0.85 | 8.32±1.10 |  | 7.93±0.34 | 6.66±1.27 | | 6.36±1.62 | **G**  **T**  **Gx** | ***  *  * |
| ***MND1*** | 4.50±0.42 | 11.25±2.33 | 4.18±0.4 | 9.97±3.17 | 3.87±0.56 | 8.01±0.69 | | 3.74±0.13 | | 12.70±2.75 | **G**  **T**  **Gx** | *  **  NS |
| ***Sphingomonas*** | 7.22±0.69 | 4.41±1.16 | 7.06±0.54 | 9.84±1.50 | 16.60±2.22 | 4.53±1.52 | | 11.76±1.15 | | 4.64±2.01 | **G**  **T**  **Gx** | ***  ***  *** |
| ***Nocardioides*** | 6.71±0.56 | 1.23±0.65 | 9.38±0.57 | 5.33±1.85 | 8.67±0.34 | 5.88±0.61 | | 10.29±0.19 | | 4.68±0.49 | **G**  **T**  **Gx** | ***  ***  *** |
| ***SWB02*** | 0.22±0.08 | 3.23±0.56 | 2.79±0.26 | 5.64±1.11 | 2.05±0.08 | 9.91±2.16 | | 2.23±0.11 | | 9.96±1.95 | **G**  **T**  **Gx** | ***  ***  *** |
| ***Ammoniphilus*** | 8.53±0.85 | 2.63±0.53 | 2.94±0.15 | 0.53±0.08 | 2.79±0.30 | 0.00±0.00 | | 3.87±0.43 | | 0.00±0.00 | **G**  **T**  **Gx** | ***  ***  *** |
| ***Blastococcus*** | 4.60±0.34 | 8.51±0.93 | 1.99±0.26 | 3.86±0.57 | 1.60±0.16 | 3.55±0.64 | | 1.82±0.19 | | 6.99±2.66 | **G**  **T**  **Gx** | ***  ***  ** |
| ***Streptomyces*** | 9.03±0.33 | 2.33±0.55 | 8.25±0.38 | 5.42±0.36 | 9.13±0.62 | 6.57±0.99 | | 9.48±1.25 | | 6.28±0.89 | **G**  **T**  **Gx** | ***  ***  ** |

(mean±sd; n=5). G, Group (which corresponds to cropping system); T, Time and GxT, interaction of Group x Time, M; Melon monocrop, MC1; Mixed intercropping, MC2; Intercropping row 1:1 melon:cowpea, MC3; Intercropping row 2:1 melon:cowpea.


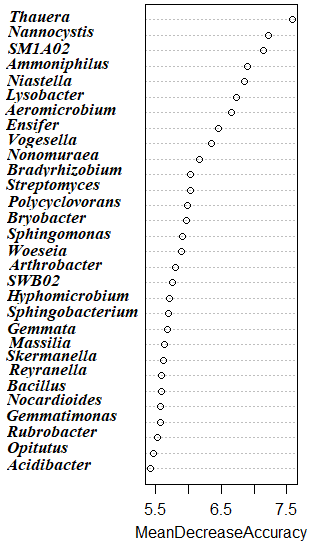


A

B


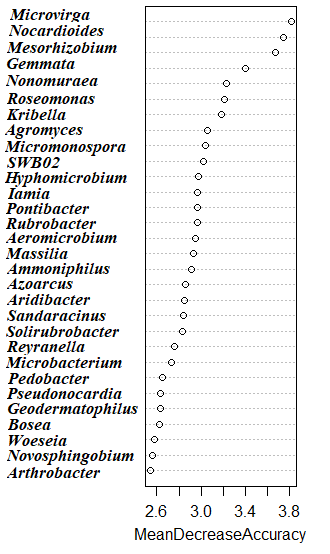


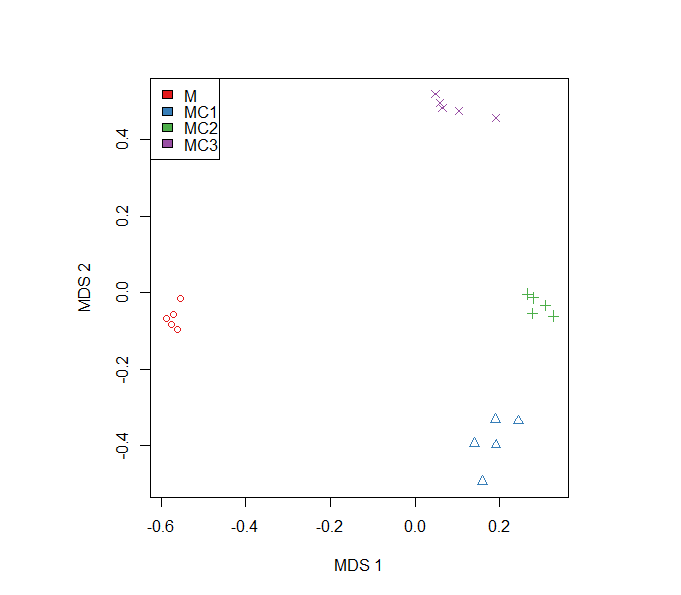

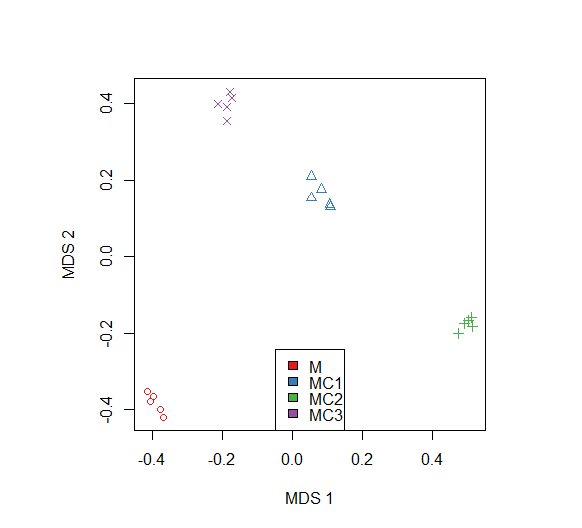


D

C

**Supplementary Fig. 4 The top 30 bacteria genera by Random Forest (RF) and multidimensional scaling (MDS) plot in the first (A, C) and third (B, D) years across the cropping systems.** M, melon monocrop; MC1, mixed intercropping; MC2, intercropping row 1:1 melon:cowpea; MC3, intercropping row 2:1 melon:cowpea.

**Supplementary Table S4. Potential metabolic pathways predicted by PICRUSt2 in different sampling times and cropping systems.**

| **Metabolic Pathway** | **First** | | | | **Third** | | | |  |  |
| --- | --- | --- | --- | --- | --- | --- | --- | --- | --- | --- |
|  | **M** | **MC1** | **MC2** | **MC3** | **M** | **MC1** | **MC2** | **MC3** |  | **P-Value** |
| **Bacterial secretion system** | 1.19±0.05 | 1.30±0.04 | 1.30±0.04 | 1.25±0.04 | 1.24±0.18 | 1.43±0.21 | 1.41±0.08 | 1.28±0.14 | **G**  **T**  **GxT** | *******  ******  **ns** |
| **Nitrogen Metabolism** | 0.70±0.02 | 0.74±0.03 | 0.75±0.07 | 0.71±0.03 | 0.70±0.14 | 0.75±0.05 | 0.77±0.03 | 0.77±9.03 | **G**  **T**  **GxT** | **ns**  ******  **ns** |
| **Energy metabolism** | 0.78±0.02 | 0.81±0.02 | 0.79±0.02 | 0.79±0.03 | 0.72±0.12 | 0.85±0.03 | 0.84±0.05 | 0.84±0.07 | **G**  **T**  **GxT** | *****  *******  **ns** |
| **Carbon fixation pathways in prokaryotes** | 1.65±0.04 | 1.75±0.09 | 1.85±0.21 | 1.63±0.21 | 1.42±0.24 | 1.86±0.38 | 1.72±0.08 | 1.65±0.22 | **G**  **T**  **GxT** | *****  **ns**  **ns** |
| **Carbon fixation in Photosyntetic** | 0.68±0.01 | 0.73±0.05 | 0.77±0.07 | 0.68±0.03 | 0.65±0.05 | 0.77±0.11 | 0.72±0.06 | 0.70±0.03 | **G**  **T**  **GxT** | ******  **ns**  **ns** |
| **TCA Cycle** | 1.33±0.02 | 1.39±0.08 | 1.37±0.03 | 1.33±0.07 | 1.09±0.18 | 0.16±0.04 | 1.37±0.06 | 1.29±0.23 | **G**  **T**  **GxT** | *******  ******  ****** |
| **Protein Export** | 1.08±0.02 | 1.13±0.03 | 1.13±0.03 | 0.90±0.44 | 0.93±0.12 | 1.24±0.33 | 1.08±0.07 | 1.01±0.12 | **G**  **T**  **GxT** | *******  *****  **ns** |
| **Transporters** | 9.75±0.25 | 10.01±0.65 | 9.95±0.71 | 10.19±0.93 | 9.13±1.25 | 11.21±2.27 | 9.65±0.26 | 9.54±0.36 | **G**  **T**  **GxT** | **ns**  **ns**  **ns** |

(mean±sd; n=5). G, Group (which corresponds to cropping system); T, Time and GxT, interaction of Group x Time, M; Melon monocrop, MC1; Mixed intercropping, MC2; Intercropping row 1:1 melon:cowpea, MC3; Intercropping row 2:1 melon:cowpea.

**Supplementary Table S5. Abundance genes from different metabolic pathways predicted by PICRUSt2 in soil samples under different cropping systems.**

| **Soil**  **Properties** | **First** | | | | **Third** | | | |  |  |
| --- | --- | --- | --- | --- | --- | --- | --- | --- | --- | --- |
|  | **M** | **MC1** | **MC2** | **MC3** | **M** | **MC1** | **MC2** | **MC3** |  | **P-Value** |
| **N-Cycling** | | | | | | | | | | |
| **nifT**  (K02593) | 301±40 | 504±189 | 419±38 | 497±127 | 535±115 | 605±100 | 178±48 | 155±46 | **G**  **T**  **GxT** | ***  *  *** |
| **nifX**  (K02596) | 326±71 | 571±123 | 459±122 | 497±127 | 544±86 | 634±15 | 169±66 | 261±91 | **G**  **T**  **GxT** | ***  ns  *** |
| **narG**  (K00370) | 4168±289 | 4715±266 | 4425±198 | 5312±444 | 5711±1219 | 8697±124 | 7507±2173 | 10382±1890 | **G**  **T**  **GxT** | ***  ***  ns |
| **nirB**  (K00362) | 11496±719 | 12313±756 | 11012±713 | 13801±1450 | 13873±497 | 22016±3240 | 18159±1157 | 12080±894 | **G**  **T**  **GxT** | *  ***  *** |
| **amoA**  (K10944) | 40±16 | 60±24 | 48±8 | 73±23 | 0±0 | 42±14 | 77±27 | 0±0 | **G**  **T**  **GxT** | ***  ***  *** |
| **amoC**  (K10946) | 86±38 | 88±21 | 83±21 | 151±93 | 0±0 | 0±0 | 312±102 | 0±0 | **G**  **T**  **GxT** | ***  ***  *** |
| **nosZ**  (K00376) | 361±88 | 664±189 | 495±123 | 611±102 | 1582±298 | 1665±578 | 1688±297 | 944±302 | **G**  **T**  **GxT** | **  ***  ** |
| **C-Cycling** | | | | | | | | | | |
| **glcD**  **(**K00104) | 27553±1463 | 29059±1619 | 27484±2030 | 33094±2516 | 38216±1943 | 42846±2511 | 39201±927 | 22599±1632 | **G**  **T**  **GxT** | ***  ***  *** |
| **xylA**  **(**K01805) | 7156±535 | 7652±447 | 7273±725 | 8434±490 | 10275±1492 | 11819±1393 | 11220±967 | 6301±523 | **G**  **T**  **GxT** | ***  ***  *** |
| **Alpha-amylase**  **(**K07405) | 454±72 | 565±64 | 512±78 | 858±124 | 805±46 | 1387±345 | 1494±77 | 616±74 | **G**  **T**  **GxT** | ***  ***  *** |
| **glucokinase**  (K00845) | 36511±2152 | 41187±2617 | 40189±1369 | 42574±3211 | 50202±3289 | 60212±3152 | 56267±1628 | 32137±3090 | **G**  **T**  **GxT** | ***  ***  *** |
| **Piruvate kinase**  (K00873) | 30246±1251 | 36699±1491 | 31529±3541 | 40748±3772 | 42259±5215 | 49582±3658 | 47227±2001 | 26778±1382 | **G**  **T**  **GxT** | ***  ***  *** |

(mean±sd; n=5). G, Group (which corresponds to cropping system); T, Time and GxT, interaction of Group x Time, M; Melon monocrop, MC1; Mixed intercropping, MC2; Intercropping row 1:1 melon:cowpea, MC3; Intercropping row 2:1 melon:cowpea.
